# Altered electrophysiology mechanism related to inhibitory control in adults with insomnia

**DOI:** 10.3389/fneur.2023.1271264

**Published:** 2023-11-22

**Authors:** Xiaobin Ding, Liang He, Xicong Geng, Xuan Zhao, Zijing He, Xiangzi Zhang

**Affiliations:** School of Psychology, Northwest Normal University, Lanzhou, China

**Keywords:** insomnia disorder, inhibitory control, two-choice oddball task, color-word Stroop task, event-related potential

## Abstract

**Background:**

Insomnia disorder (ID), one of the most common psychophysiological disorders, can cause a serious burden on the individual's work and academic performance. Cognitive dysfunction often exists in patients with insomnia, which negatively affects their living quality. Inhibitory control (IC), as a vital cognitive function, allows individuals to suppress attention, behavior, or thoughts that are irrelevant to the task, so as to effectively adapt to the current goal. The earlier studies on the inhibitory control of insomnia patients predominantly used subjective scales for evaluation and that can have drawbacks because they don't provide an objective assessment.

**Methods:**

In order to investigate the inhibitory control function of insomniacs, this research subdivides inhibitory control into response inhibition and conflict inhibition. The response inhibition and conflict inhibition capacities of insomniacs were evaluated using the two-choice oddball task and the color-word stroop task, and accordingly the association between insomnia disorder and inhibitory control capacity as well as its cognitive neural mechanism was able to be examined.

**Results:**

Behavioral results finding, insomniacs conducted the two-choice oddball test and the color-word stroop task with lower accuracy and slower reaction times when compared to healthy sleepers. ERP results finding, when performing the two-choice oddball task, the P3 amplitude of the insomniacs was significantly lower than that of healthy sleepers while there was no significant difference between the two groups' N2 amplitudes. At the same time, when completing the color-word stroop task, the insomniacs' N450 amplitude was significantly lower than that of healthy sleepers.

**Discussion:**

The above findings suggest that in response inhibition tasks, insomniacs may have weaker motor inhibition abilities, and similarly perform weaker conflict monitoring abilities in conflict inhibition tasks, which indicates that insomniacs' inhibitory control is impaired compared to that of healthy sleepers. This study thus relates to the finding at the electrophysiological level that there is a certain correlation between insomnia and a decline in inhibitory control ability, which may suggest that improving inhibitory control function in patients with insomnia is a clinically significant and worthwhile area of adjuvant treatment.

## 1 Introduction

Currently, more and more people are struggling with insomnia. Insomnia disorder (ID) is one of the most prevalent and common psychophysiological disorders (1). According to the International Classification of Sleep Disorders-Third Edition (ICSD-3) (2), ID refers to a disease in which patients suffer from persistent loss of sleep duration, loss of sleep integrity, difficulty falling asleep and waking up, loss of sleep quality, and related impairment of body function even when sleep opportunities and environments are adequate. Individuals with insomnia often have cognitive impairment and its influencing factors, which seriously affect their quality of life. Inhibitory control is a very important cognitive function that allows individuals to suppress attention, behavior, or thoughts unrelated to the task and to adapt flexibly to the current goal (3). Harvey (4) proposed a maintenance cognitive model of insomnia. Insomnia often involves worries about sleep and the consequences of lack of sleep, which can trigger spontaneous awakening and emotional distress or anxiety, which can lead to cognitive distortions about sleep deprivation and counterproductive behaviors. The insomnia disorder group attempts to control or shut down uncontrollable, unpleasant presleep thoughts through inhibition, which paradoxically stimulates unnecessary cognitive activity and prolongs sleep latency. As a very important executive function, inhibitory control allows individuals to suppress thoughts or actions irrelevant to the current task and flexibly adapt individual psychology and behavior to the current goal (5).

Currently, research on executive control in ID remains controversial. A review of the literature on executive function found that most studies found no impairment of executive function in primary insomnia (6). This is inconsistent with neuroimaging findings of structural and functional changes in the prefrontal cortex in individuals with insomnia (7). For example, Sagaspe et al. (8) used the stop signal task to study the motor inhibition ability of patients with obstructive sleep apnea syndrome (OSAS) and psychophysiological insomnia and found that there was no difference in stop signal reaction time between psychophysiological individuals with insomnia and controls. Several other studies also did not find impaired inhibitory control in patients with insomnia (9–11). However, Covassin et al. (12) found that patients with insomnia had a prolonged stop signal reaction time, which showed impaired motor inhibition in patients with insomnia using behavioral indicators. Additionally, a study using a go/no-go task found no significant differences in reaction times and accuracy between subjects with primary insomnia and healthy controls (9). Zhao et al. (13) used the auditory stop signal paradigm coupled with event-related potentials (ERPs). The results revealed that compared to healthy sleepers (HSs), patients with insomnia presented a significantly longer stop signal reaction time and reduced P3 amplitude in patients with insomnia during successful stop trials. These suggest the impairment of motor inhibition among individuals with insomnia. A recent study (14) combined a go/no-go task and EEG to measure adolescents with insomnia. The results showed that adolescents with insomnia showed altered brain activity during inhibitory control. Therefore, studies on the relationship between ID and inhibitory control still have inconsistent results. On the one hand, most previous studies only used a single inhibitory control task, and the components of inhibitory control measured by different tasks are different. For example, the two-choice oddball task and the go/no-go task measure the response inhibition in inhibitory control, and the Stroop and Simon tasks mainly measure conflict control in inhibitory control (3, 15). On the other hand, previous studies mainly used behavioral indicators to evaluate the relationship between ID and inhibitory control, but behavioral indicators could not effectively reflect the characteristics of each stage of cognitive processing. For example, completing a go/no-go task involves multiple processes such as stimulus perception, stimulus discrimination, response selection, and response execution or response inhibition (16).

Based on this, this study uses ERP technology to investigate the differences and characteristics of inhibitory control ability between healthy sleepers (HSs) and insomnia disorders (IDs) through two-choice oddball (response inhibition) and color-word Stroop (conflict inhibition) tasks. Yuan et al. (17) modified the classical oddball task into the two-choice oddball task through many studies. They found that the two-choice oddball task could be widely used in the sensitive measurement and evaluation of the intervention of inhibitory control ability and its influencing factors. Many researchers have used the two-choice oddball task to assess response suppression in various fields, such as individual differences (17, 18), emotion (19), and smoking addiction (13). Therefore, the two-choice oddball task can be widely used in sensitive measurement and intervention evaluation of the control ability of behavioral inhibition and its influencing factors. In terms of behavioral indicators, the correct rate of deviant stimuli decreases less than that of standard stimuli, indicating stronger behavioral inhibition and control ability of the subjects (20). In terms of EEG components, the two-choice oddball task will induce more obvious N2 and P3 components, which are distributed in the frontal-central area (19). For example, the go/no-go task also reflected response inhibition. The effect of N2 no-go is considered related to conflict monitoring during response inhibition and is generally measured by the amplitude of N2 induced by the no-go condition or the difference amplitude of N2 (N2d) of the no-go minus the go condition, and the no-go N2 or the larger the amplitude of N2d may reflect stronger conflict monitoring capabilities (21–24). Motor inhibition during response inhibition is connected to the no-go P3 effect. The no-go P3 (P3d) was of bigger amplitude; the stronger the motor inhibition ability may be indicated. This is generally measured by the amplitude of P3 generated by the no-go condition, or the difference amplitude of P3 (P3d) of the no-go condition minus the go condition (25–28). One advantage of the two-choice oddball task is that since subjects need to perform behavioral responses to both standard and deviant stimuli, the ERP under the deviant stimulus minus the ERP under the standard stimulus can eliminate the interference of factors related to action preparation and action execution. This difference wave is a direct indicator of response inhibition (29). The Stroop task is one of the paradigms for examining conflict inhibition, which includes two conditions: conflict (word meaning and word color mismatch) and non-conflict (word meaning and word color match) conditions (30). The Stroop task tends to induce more pronounced N450 (31–33). N450 generally appears in the time window of 300~600 ms after stimulus presentation, peaks at 450 ms, and is distributed in the frontal-central area. Inconsistent conditions will induce greater N450 amplitudes than consistent conditions (31, 34). The N450 component is related to conflict monitoring in the conflict control process, and a larger difference wave of N450 (inconsistent condition minus consistent condition) may reflect a stronger conflict monitoring ability (33, 35). The reason we use difference waves is to minimize ERP differences between groups that may arise from differences in skull thickness, head tissue conductivities, and head/brain geometries (36, 37). Moreover, this rationale is also applicable to N2, P3, and N450.

Taken together, through the use of the two-choice oddball task, the color-word Stroop task, and ERPs, the main aim of our study is to address an important question: whether individuals with insomnia would have inhibitory control deficits and the potential electrophysiological mechanism of inhibitory control abilities. Based on previous studies, we hypothesized that, compared to healthy sleepers, insomnia disorders would show impaired inhibitory control at both the behavioral and electrophysiological levels, such as the time domain. At the behavioral level, the reaction time of IDs was slower than that of HSs. The correct rate of IDs was also lower than that of HSs. At the electrophysiological level, if HSs have stronger conflict monitoring or movement inhibition ability in response inhibition, then their N2 difference wave or P3 difference wave amplitude is greater than that of IDs in the two-choice oddball task. If HSs have stronger conflict monitoring or conflict resolution capabilities in conflict inhibition, then their N450 difference wave amplitude in the Stroop task is greater than that of the IDs.

## 2 Methods

### 2.1 Participants and experimental design

According to the G^*^Power software (38), the sample size required for the study was estimated in advance (f = 0.25, α = 0.05, power = 0.80), and it was found that the minimum required sample size was 56, and each group had a minimum of 28 people. Therefore, the sample size of this experiment meets this requirement. In total, 33 healthy sleepers (HSs, 16 men; mean age = 23.27 years, SD = 2.64) and 34 insomnia disorders (IDs, 9 men, mean age = 23.12 years, SD = 2.74) participated in this experiment. Advertisements were used to recruit all members of the community. Each participant received a complete physical examination along with laboratory tests and psychological evaluations. Pittsburgh Sleep Quality Index (PSQI) (39), Beck Anxiety Inventory (BAI) (40), Insomnia Severity Index (ISI) (41), and Multidimensional Fatigue Inventory (MFI) (42) were completed to assess sleep quality and emotion state. An experienced mental health physician used unstructured interviews to diagnose the 34 patients with insomnia. These insomnia patients met the following conditions: (1) age between 18 and 35; (2) meeting the definition of insomnia disorders of the International Classification of Sleep Disorders-Third Edition (they include a report on sleep initiation or maintenance problems, adequate opportunity and circumstances to sleep, and daytime consequences); (3) ISI score ≥ 15 and PSQI score ≥ 7; (4) no congenital or acquired mental and physical diseases; (5) no additional sleep problems (such as sleep-related movement disorders, hypersomnia, or abnormal sleep); (6) difficulty sleeping for at least 3 months (including difficulty initiating sleep, difficulty maintaining sleep, early morning awakening, or a non-restorative sleep); and (7) participants did not take psychotropic drugs 2 weeks before and during the experiment. All the HSs met the following criteria: (1) age between 18 and 35 years; (2) participants do not have difficulty initiating sleep, difficulty maintaining sleep, or early morning awakening; (3) good sleep quality and no history of alternating day and night work; (4) no psychoactive drugs, tea, and coffee were taken during the participation process, 2 weeks before the experiment, and during the experiment; (5) ISI score <15, PSQI score <7, BAI standard score <50; and (6) no neurological or mental disease.

Our study used a two-choice oddball task and a color-word Stroop task, and all subjects were tested on both tasks. Half of the participants performed the two-choice oddball task first, and the other half of the participants performed the color-word Stroop task. The two tasks are counterbalanced between participants. This study received clearance from the School of Psychology Scientific Research Ethics Council at Northwest Normal University (ethical approval No.: 20220037) and was also registered with the Chinese Clinical Trial Registry (registration No.: ChiCTR2200061186). This experiment complies with the Declaration of Helsinki. The volunteers signed the informed consent form prior to the experiment after being informed of the setup and method.

### 2.2 Experimental paradigm: two-choice oddball task

**Figure 1:**
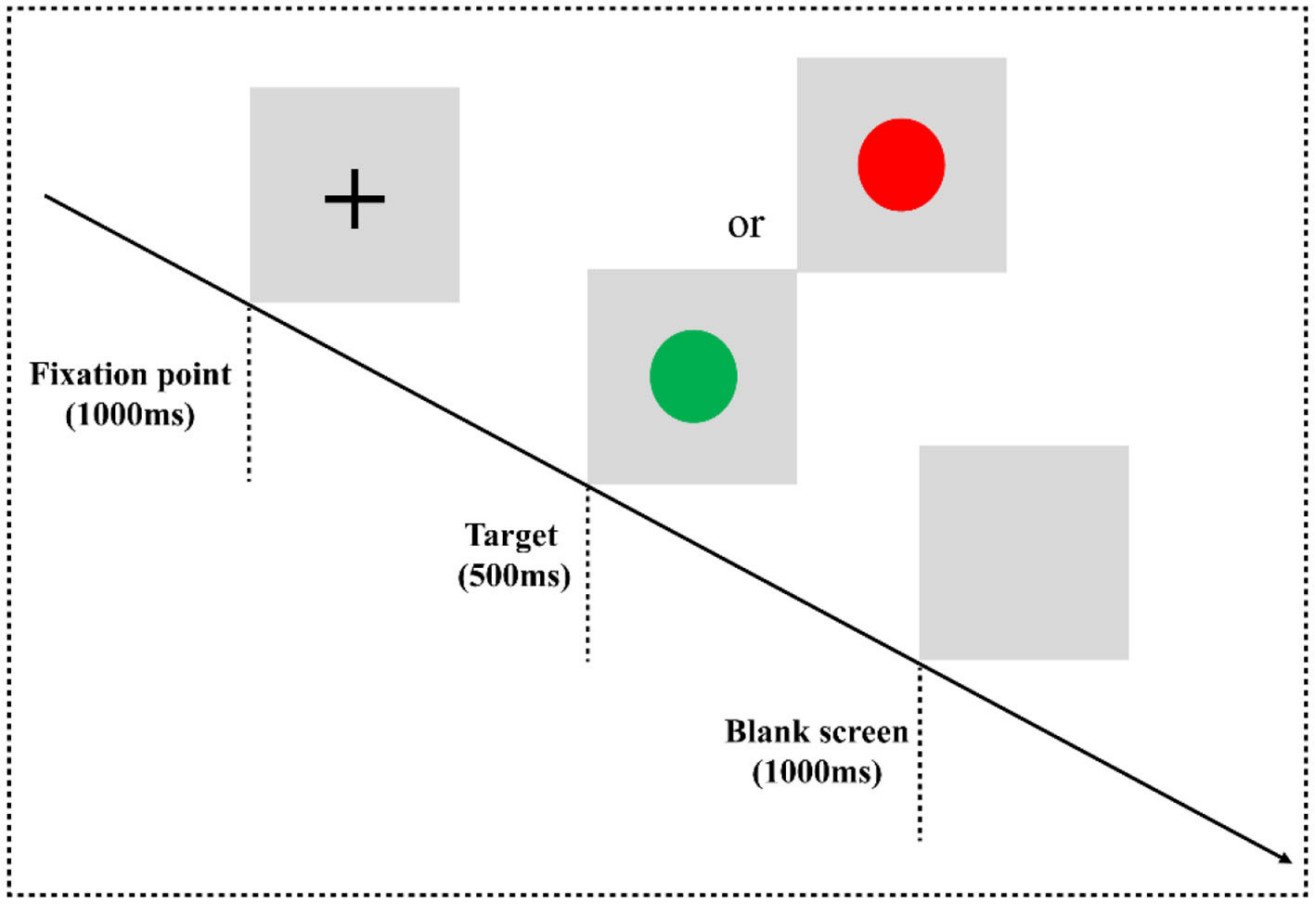
Two-choice oddball task flowchart. Experimental paradigm for the two-choice oddball task. E-prime 3.0 software was used for programming. The experiment consisted of three blocks, and each block contained 100 trials, in which the standard stimulus “red circle” appeared 75 times, and the deviant stimulus “green circle” appeared 25 times. All trials are arranged in a random manner. There is a rest period after each block, the duration of the rest period is controlled by the subjects themselves, and the whole task takes approximately 10 min.

In the two-choice oddball task (TCOT) (20) (Figure 1), the experimental materials were divided into two categories: standard stimulus and deviant stimulus (75 vs. 25%). The experimental stimuli were circles, including “red” and “green,” where the “red circle” was the standard stimulus and the “green circle” was the deviant stimulus, presented on a gray background. The experimental equipment was a Dell computer, and the keyboard was used for key response. After entering the laboratory, the subjects were informed of the purpose, requirements, and precautions of the experiment. They were asked to put on an electrode cap, sit on a chair 80 cm away from the monitor, and look at the center of the screen. At the beginning of the experiment, they were instructed that a fixation point “+” would appear at the center of the computer screen for 1,000 ms, then a stimulus would be presented for 500 ms (a red circle and a green circle would appear randomly), and finally, the screen would be empty for 1,000 ms. When given the stimuli, the subjects were required to carefully watch the stimuli and respond quickly and accurately using the categorizing keys: when subjects saw the standard stimuli, they pressed the “F” key, and when they saw the deviant stimuli, they pressed the “J” key.

### 2.3 Experimental paradigm: color-word Stroop task

**Figure 2:**
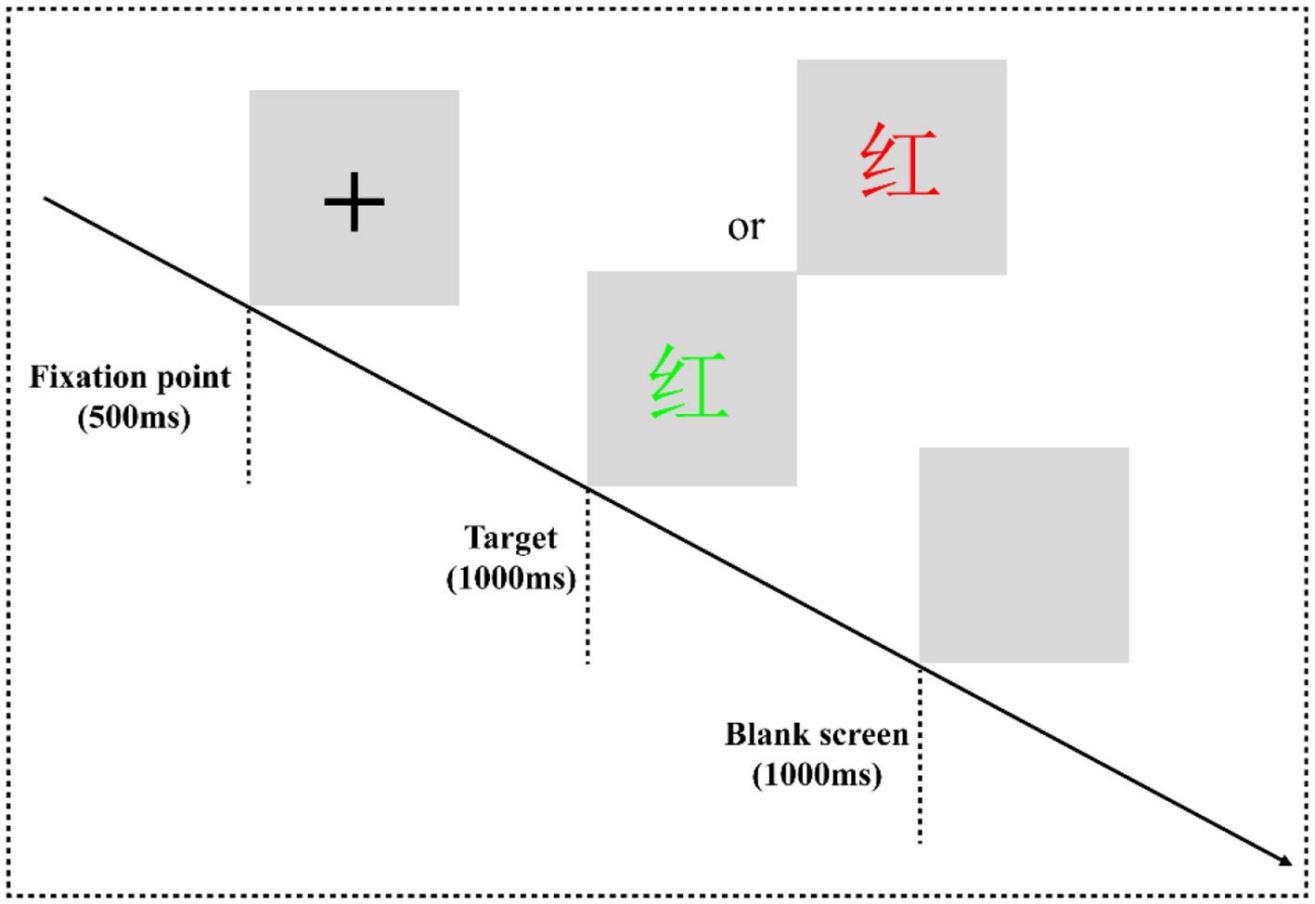
Color-word Stroop task flowchart. The experimental program is divided into one practice block and four formal experiment blocks. In the practice block, to make the subjects familiar with the key-press rules and the experiment process, the formal experiment can only be entered when the correct rate of practice reaches more than 85%. There are 16 trials in the practice block, consisting of 8 consistent trials and 8 inconsistent trials. Each block of the formal experiment has 64 trials, consisting of 32 consistent trials and 32 inconsistent trials. The formal experiment has a total of 256 trials, and all trials are arranged in a pseudorandom manner.

In the classic color-word Stroop task (CWST) (Figure 2), the words “red” and “green” written in two colors of “red” and “green” are randomly presented to form a consistent condition and an inconsistent condition. For example, “red” written in red is a consistent condition, and “red” written in other colors means inconsistent conditions. The experimental equipment was a Dell computer, and the keyboard was used for key responses. After entering the laboratory, the subjects were informed of the purpose, requirements, and precautions of the experiment. They were asked to put on an electrode cap, sit on a chair 80 cm away from the monitor, and look at the center of the screen. At the beginning of the experiment, they were instructed that a black fixation point “+” would be presented on the gray screen for 500 ms, and then a stimulus would be presented for 1,000 ms, which would disappear immediately after a response is provided. Finally, the screen would be empty for 1,000 ms, and then the next trial would begin. During the experiment, the subjects were asked to always respond to the color of the word. If the word color was red, they would have to press the “F” key on the keyboard with their left index finger to respond. If the color of the word is green, they would have to press the “J” key on the keyboard with the right index finger to react.

### 2.4 Electroencephalogram recording

A 64-channel amplifier (Advanced Neuro Technologies, ANT, Enschede, The Netherlands) was used to collect electroencephalogram (EEG) signals, and scalp electrodes were positioned according to the International 10–20 system. The online reference electrode was CPz. The resistance of each electrode point was kept below 10 kΩ. The sampling frequency was 1,000 Hz, and the electrical signals were recorded at that frequency with an online 0.01–100-Hz bandpass filter that included a 50-Hz notch filter.

### 2.5 EEG data preprocessing

EEGLAB v2021.0 and ERPLAB v8.30 were used to process EEG data offline in MATLAB (version: 2022a, MathWorks Inc., Natick, USA). Re-referencing was done using the average of the left and right mastoids. We filtered the data using ERPLAB, and the bandpass was set at 0.1~30 Hz. The baseline was 200 ms prior to the stimulus presentation, and the analysis lasted for 1,000 ms. The 200 ms prestimulus time interval served as the basis for the baseline correction. Ocular, muscular, and other artifact-containing epochs were discovered and excluded from further analysis. According to scalp maps and activity profiles of subjects, this study also employed an independent component analysis (ICA) to eliminate residual EOG artifacts. During the preprocessing of EEG data, data from subjects whose effective trials were <50% were eliminated. Therefore, in the two-choice oddball task and the color-word Stroop task, the ERP results are based on 30 HSs and 32 IDs.

### 2.6 Statistical analysis

For statistical analysis, SPSS 27.0 software (IBM, Armonk, NY, USA) was utilized. In behavioral tasks, we investigated whether the reaction time or the correct rate caused by different conditions differed between the HS participants and the ID participants. The groups (HSs and IDs) were used as the factor between the subjects, and the stimulus types (standard vs. deviant stimuli or consistency vs. inconsistency) were used as the factor within subjects. A mixed measures ANOVA was used to test whether there were any main effects or interactions between the factors. For the ERP components, we used 2 (stimulus types: standard stimulus, deviant stimulus, or consistency, inconsistency) × 2 (groups: IDs and HSs) × 4 (electrode points: Fz, FCz, Cz, and Pz) mixed measures ANOVA to discuss the differences between the two groups of subjects. The Greenhouse–Geisser approach was used to adjust the degrees of freedom of the *F*-ratio and the *p*-value for the main effect. If there was a significant interaction, a simple effects analysis was used. Based on previous studies (17–19, 32), four electrode points were selected for the data analysis: Fz, FCz, Cz, and Pz. The details are as follows: The average amplitude of the N2 and P3 and the difference wave components in the two-choice oddball task were analyzed, where the time window of the N2 (N2 difference wave) component was 220~280 ms (18) after stimulus presentation. The time window of the P3 (P3 difference wave) component was 350~450 ms (17, 19) after stimulus presentation. Next, the average amplitude of the N450 and the difference wave component in the color-word Stroop task were analyzed. The time window of the N450 (N450 difference wave) component was 400~500 ms (43) after stimulus presentation. The time windows were selected at the time series with the greatest amplitude.

## 3 Results

### 3.1 Demographic and clinical characteristics

Table 1 summarizes the demographic information and scale scores of the two groups of subjects, and an independent sample *t*-test is performed on the data of the two groups of subjects. Independent sample *t*-test results showed that the ISI, PSQI, MFI, and BAI scores of ID subjects were significantly higher than those of the HS subjects. When compared to the HS subjects, the ID subjects reported less restful sleep and might be more tired during the day.

**Table 1:** Demographic and clinical characteristics of the IDs and HSs.

	**HS (*****n*** = **34)**		**ID (*****n*** = **33)**		**Statistical analysis**	
	***M***	***SD***	***M***	***SD***	***t***	***p***
**Demographic data**						
Age (years)	23.27	2.64	23.12	2.74	0.236	0.814
Gender (men: women)	16 : 17		9 : 25		-	-
Education level (year)	15.62	1.80	15.74	2.06	−0.24	0.810
**Clinical data**						
ISI	10.91	2.57	22.50	4.54	−12.91	**< 0.001**
PSQI	3.64	1.95	10.12	3.04	−10.35	**< 0.001**
MFI	40.18	13.08	63.00	13.65	−6.98	**< 0.001**
BAI	29.39	5.95	42.74	13.81	−5.16	**< 0.001**

*M*, mean; *SD*, standard deviation; ISI, Insomnia Severity Index; PSQI, Pittsburgh Sleep Quality Index; BAI, Beck Anxiety Inventory; MFI, Multidimensional Fatigue Inventory.

### 3.2 Behavioral results

**Figure 3:**
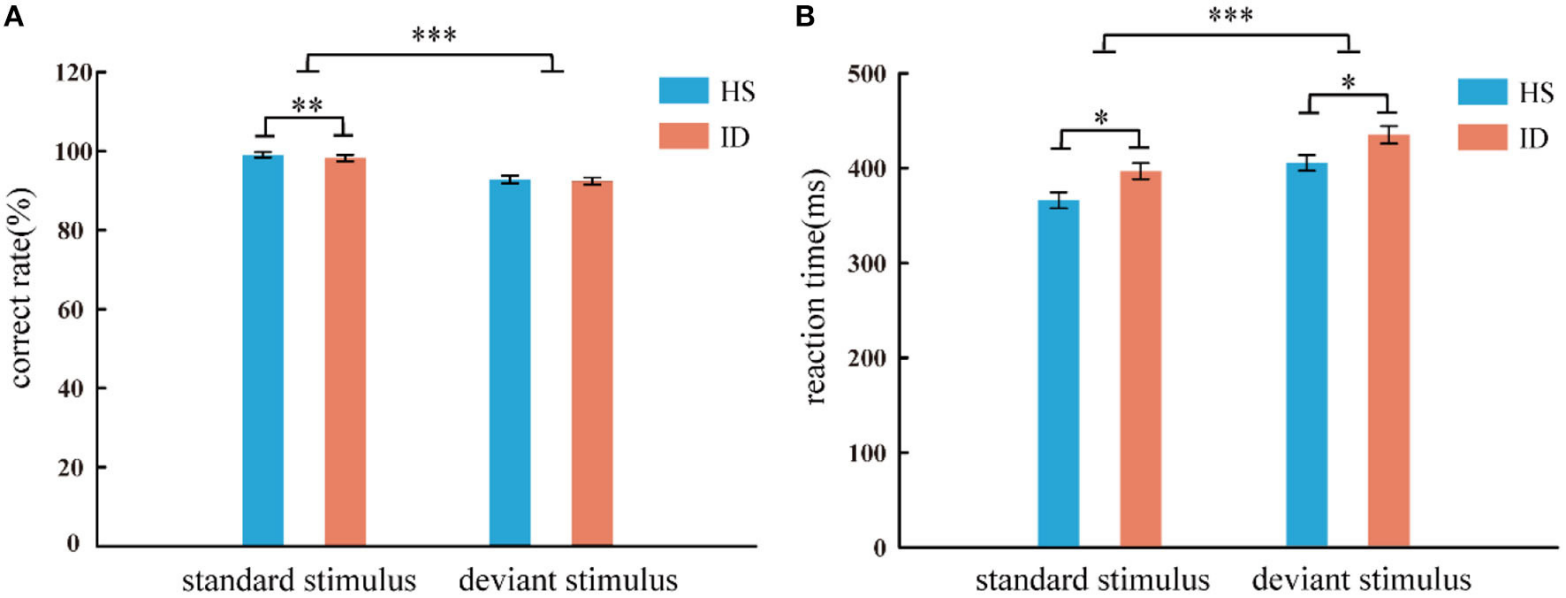
Correct rate **(A)** and reaction time **(B)** of two groups of subjects under different conditions (standard stimulus and deviation stimulus). Error bars: ± SEM, *: *p* < 0.05, **: *p* < 0.01, ***: *p* < 0.001. One subject in the ID group misinterpreted the instructions, resulting in a correct rate lower than 50%, and the data had to be excluded. Therefore, the behavioral results are based on 33 HS subjects and 33 ID subjects.

In the two-choice oddball task, the analysis of the variance of the correct rate shows that the main effect of the stimulus types was significant [*F*_(1, 64)_ = 91.753, *p* < 0.001, η2 p = 0.598]. The correct rate of subjects in the standard stimulus (*M* ± *SE*: 0.99 ± 0.01) was higher than that under the deviant stimulus (*M* ± *SE*: 0.93 ± 0.05). However, the main effect of the groups was not significant [*F*_(1, 64)_ = 0.625, *p* = 0.432] nor was the interaction between the two variables [*F*_(1, 64)_ = 0.103, *p* = 0.750]. An independent samples *t*-test showed that under the standard stimulus, the correct rate of the HS group (99.02%) was significantly higher than that of the ID group (98.25%) [*t*_(64)_ = 2.687, *p* = 0.009]. However, under the deviant stimulus, there was no significant difference between the correct rate (92.81%) of the HS group and the correct rate (92.44%) of the ID group [*t*_(64)_ = 0.277, *p* = 0.782], as presented in Figure 3A.

In the two-choice oddball task, an analysis of variance of reaction time shows the main effect of stimulus types was significant [*F*_(1, 64)_ = 117.038, *p* < 0.001, η2 p = 0.646]. Subjects' reaction time under the standard stimulus (381.56 ms) was faster than that of the deviant stimulus (420.10 ms). The main effect of the groups was significant [*F*_(1, 64)_ = 6.786, *p* = 0.011, η2 p = 0.096]. The average reaction time of the HS group (385.72 ms) was faster than that of the ID group (415.94 ms). However, the interaction between the two variables was not significant [*F*_(1, 64)_ = 0.029, *p* = 0.866]. An independent sample *t*-test showed that under the standard stimulus, the reaction time of the HS group (366.15 ms) was significantly shorter than that of the ID group (396.97 ms) [*t*_(64)_ = −2.598, *p* = 0.012]. Under the deviant stimulus, the reaction time of the HS group (405.29 ms) was also significantly shorter than that of the ID group (434.90 ms) [*t*_(64)_ = −2.389, *p* = 0.020], as presented in Figure 3B.

**Figure 4:**
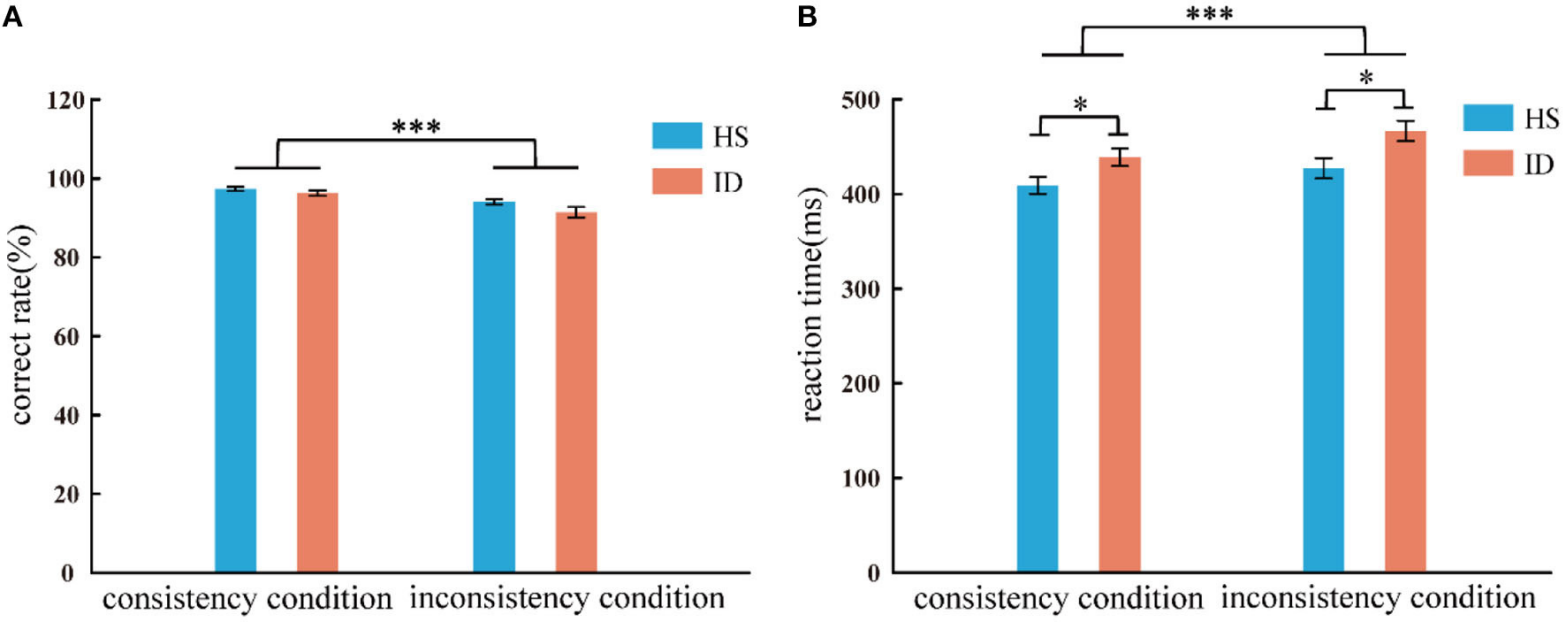
Correct rate **(A)** and response time **(B)** of the two groups of subjects under different conditions (consistency and inconsistency). Error bars: ±SEM, *p < 0.05, ***p < 0.001.

In the color-word Stroop task, the analysis of variance of correct rate shows that the main effect of the conditions was significant [*F*_(1, 65)_ = 50.011, *p* < 0.001, η2 p = 0.435]. The correct rate of subjects in the consistent conditions (M ± SE: 0.97 ± 0.03) was higher than in the inconsistent conditions (*M* ± *SE*: 0.93 ± 0.06). The main effect of the groups was not significant [*F*_(1, 65)_ = 3.098, *p* = 0.083]. The interaction between the two variables was not significant [*F*_(1, 65)_ = 1.763, *p* = 0.189]. An independent sample *t*-test showed that under consistent conditions, the correct rate of the HS group (97.35%) was not significantly different from that of the ID group (96.28%) [*t*_(65)_ = 1.373, *p* = 0.174]; Under inconsistent conditions, the correct rate of the HS group (94.03%) was not significantly different from that of the ID group (91.43%) [*t*_(65)_ = 1.741, *p* = 0.086], as presented in Figure 4A.

In the color-word Stroop task, the analysis of variance of reaction time shows that the main effect of conditions was significant [*F*_(1, 65)_ = 104.498, *p* < 0.001, η2 p = 0.617]. Subjects' reaction time under consistent conditions (424.16 ms) was faster than that under inconsistent conditions (447.26 ms). The main effect of the groups was significant [*F*_(1, 65)_ = 6.208, *p* = 0.015, η2 p = 0.087], and the average reaction time of the HS group (418.19 ms) was faster than that of the ID group (452.73 ms). Furthermore, the interaction between the conditions and the groups was significant [*F*_(1, 65)_ = 4.657, *p* = 0.035, η2 p = 0.067]. Further simple effects analysis showed that the reaction time of the HS group (409.10 ms) was significantly faster than that of the ID group (438.78 ms) under consistent conditions [*F*_(1, 65)_ = 5.324, *p* = 0.024, η2 p = 0.076]. Under inconsistent conditions, the reaction time of the HS group (427.27 ms) was significantly faster than that of the ID group (466.67 ms) [*F*_(1, 65)_ = 6.777, *p* = 0.011, η2 p = 0.094], as presented in Figure 4B.

### 3.3 Two-choice oddball task event-related potential results

**Figure 5:**
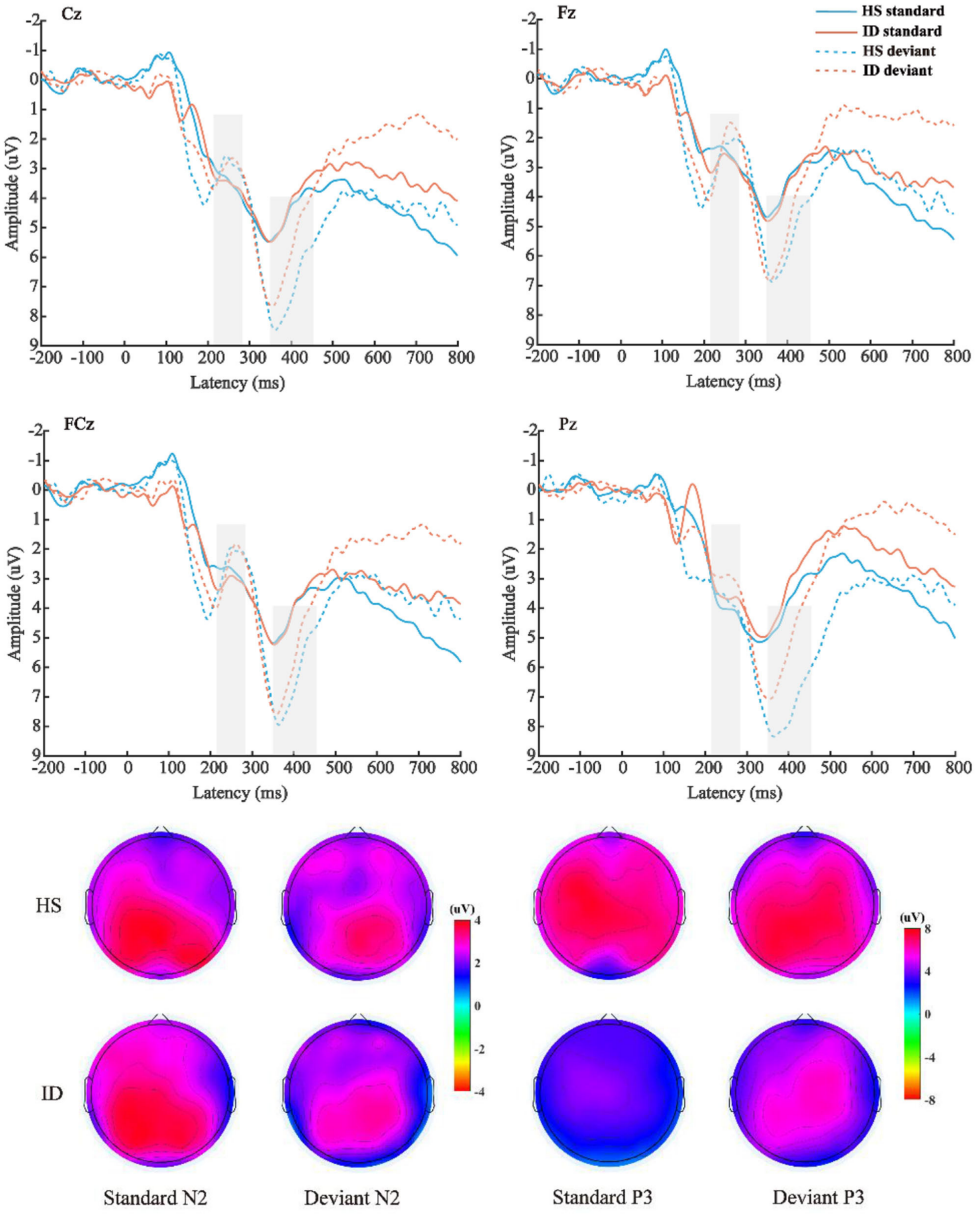
**Top**: The average ERP waveforms of the scalp positions of Cz, Fz, FCz, and Pz of the subjects in the HS group and the ID group under the two stimulation types, respectively. **Bottom**: Scalp voltage topography of the N2 and P3 components of the HS group and the ID group under the two types of stimulation, respectively.

The average amplitude of the N2 component was analyzed with 2 (stimulus types: standard stimulus and deviant stimulus) × 2 (groups: HS group and ID group) × 4 (electrode points: Fz, FCz, Cz, and Pz) mixed measures variance analysis. The results are shown in Figure 5; the main effect of the electrode points was significant [*F*_(3, 180)_ = 18.302, *p* < 0.001, η2 p = 0.234], and the amplitude of the Pz electrode point (*M* ± *SE*: 3.62 ± 0.40 μV) was significantly larger than that of the Cz electrode point (*M* ± *SE*: 3.21 ± 0.42 μV), FCz electrode points (*M* ± *SE*: 2.65 ± 0.42 μV), and the amplitudes of the Fz electrode points (*M* ± *SE*: 2.45 ± 0.38 μV). However, neither the main effect of the stimulus types [*F*_(1, 60)_ = 2.198, *p* = 0.143] nor the main effect of the groups was significant [*F*_(1, 60)_ = 0.008, *p* = 0.930].

The mean amplitude of the P3 component was analyzed with 2 (stimulus types: standard stimulus and deviant stimulus) × 2 (groups: HS group and ID group) × 4 (electrode points: Fz, FCz, Cz, and Pz) mixed measures variance analysis. The results are shown in Figure 5; the main effect of stimulus types was significant [*F*_(1, 60)_ = 44.292, *p* < 0.001, η2 p = 0.425], and the amplitude (*M* ± *SE*: 6.12 ± 0.52 μV) under the deviant stimulus was significantly larger than under the standard stimulus (*M* ± *SE*: 3.93 ± 0.47 μV). The main effect of the electrode points is significant [*F*_(3, 180)_ = 9.402, *p* < 0.001, η2 p = 0.135], and the amplitude of the Cz electrode point (*M* ± *SE*: 5.44 ± 0.47 μV) is significantly larger than that of the FCz electrode point (*M* ± *SE*: 5.09 ± 0.49 μV), Pz electrode point (*M* ± *SE*: 5.06 ± 0.47 μV), and the amplitude of the Fz electrode point (*M* ± *SE*: 4.50 ± 0.45 μV). However, the main effect of the groups was not significant [*F*_(1, 60)_ = 0.568, *p* = 0.454]. There was no significant interaction between the stimulus types and groups [*F*_(1, 60)_ = 2.848, *p* = 0.097, η2 p = 0.045].

Furthermore, the interaction between the electrode points and the groups was significant [*F*_(3, 180)_ = 3.773, *p* = 0.028, η2 p = 0.059]. A further simple effect analysis showed that the average amplitudes of the HS group at the Fz electrode point (*M* ± *SE*: 4.63 ± 0.65 μV) were greater than that of the ID group (*M* ± *SE*: 4.38 ± 0.63 μV). At the FCz electrode site, the average amplitudes of the HS group (*M* ± *SE*: 5.30 ± 0.71 μV) were greater than that of the ID group (*M* ± *SE*: 4.88 ± 0.68 μV). At the Cz electrode site, the average amplitudes of the HS group (*M* ± *SE*: 5.83 ± 0.72 μV) were greater than that of the ID group (*M* ± *SE*: 5.05 ± 0.69 μV). At the Pz electrode site, the average amplitudes of the HS group (*M* ± *SE*: 5.74 ± 0.68 μV) were greater than that of the ID group (*M* ± *SE*: 4.38 ± 0.66 μV).

**Figure 6:**
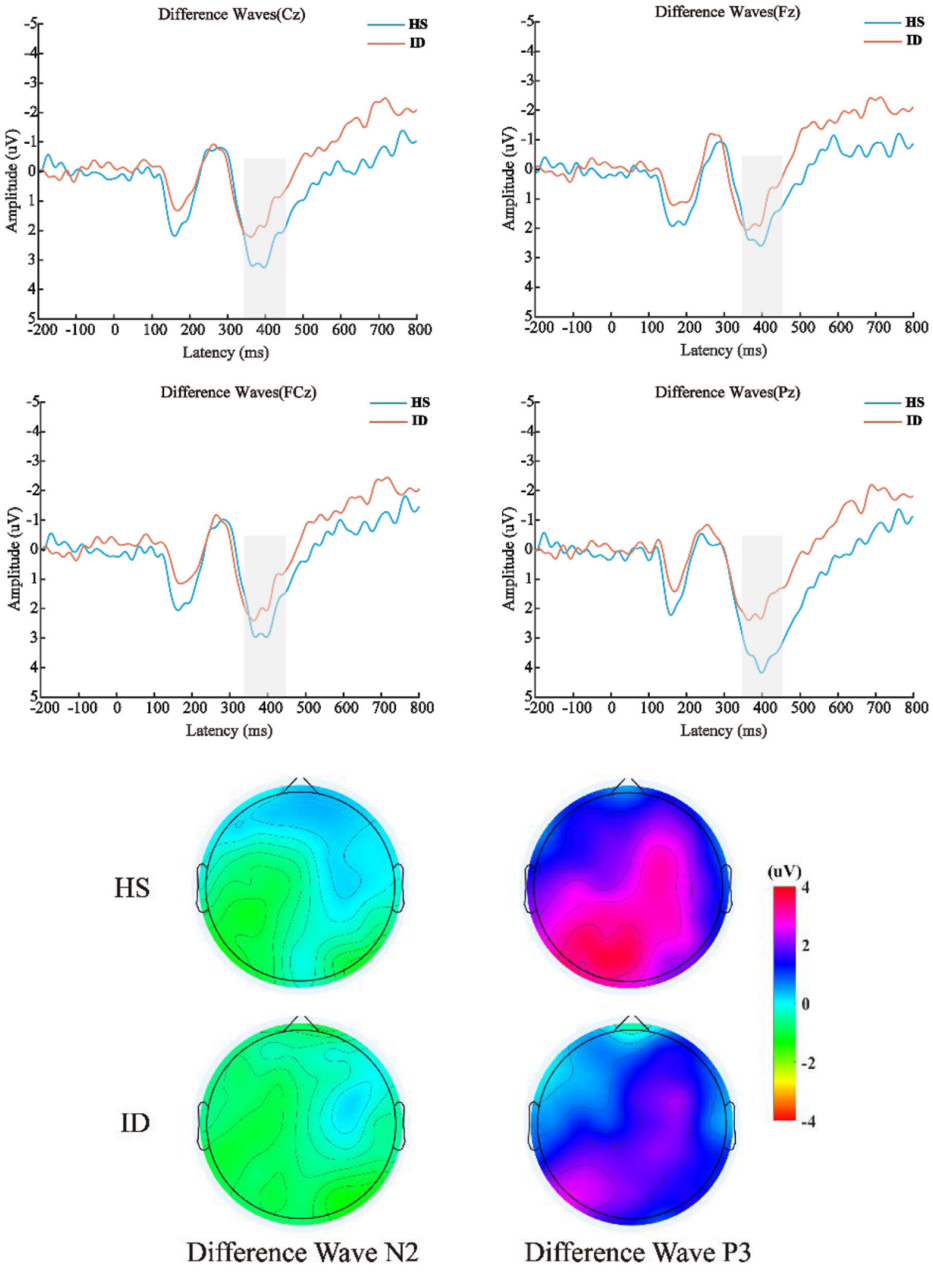
**Top**: The difference wave (deviation stimulus minus standard stimulus) average ERP waveforms of the HS group and ID group at the scalp positions of Cz, Fz, FCz, and Pz, respectively. **Bottom**: Differential wave scalp voltage topography maps of the HS group and ID group under N2 and P3 components, respectively.

The 2 (groups) × 4 (electrode points) ANOVA was performed on the difference wave, and the results are shown in Figure 6. On the component of the difference wave N2, the main effect of the electrode points was not significant [*F*_(3, 180)_ = 1.610, *p* = 0.197, η2 p = 0.026]. The main effect of the groups was not significant [*F*_(1, 60)_ = 0.187, *p* = 0.667, η2 p = 0.003]. There was no significant interaction between the electrode points and the groups [*F*_(3, 180)_ = 1.253, *p* = 0.292, η2 p = 0.020]. On the component of the difference wave P3, the main effect of the electrode points was significant [*F*_(3, 180)_ = 10.871, *p* < 0.001, η2 p = 0.153], and the amplitude of the Pz electrode point (*M* ± *SE*: 2.81 ± 0.32 μV) was significantly larger than that of the Cz electrode point (*M* ± *SE*: 2.17 ± 0.36 μV), FCz electrode point (*M* ± *SE*: 2.05 ± 0.35 μV), and the amplitude of the Fz electrode point (*M* ± *SE*: 1.74 ± 0.37 μV). The main effect of the groups was not significant [*F*_(1, 60)_ = 2.848, *p* = 0.097, η2 p = 0.045]. There was no significant interaction between the electrode points and the groups [*F*_(3, 180)_ = 2.864, *p* = 0.060, η2 p = 0.046].

### 3.4 Color-word Stroop task event-related potential results

**Figure 7:**
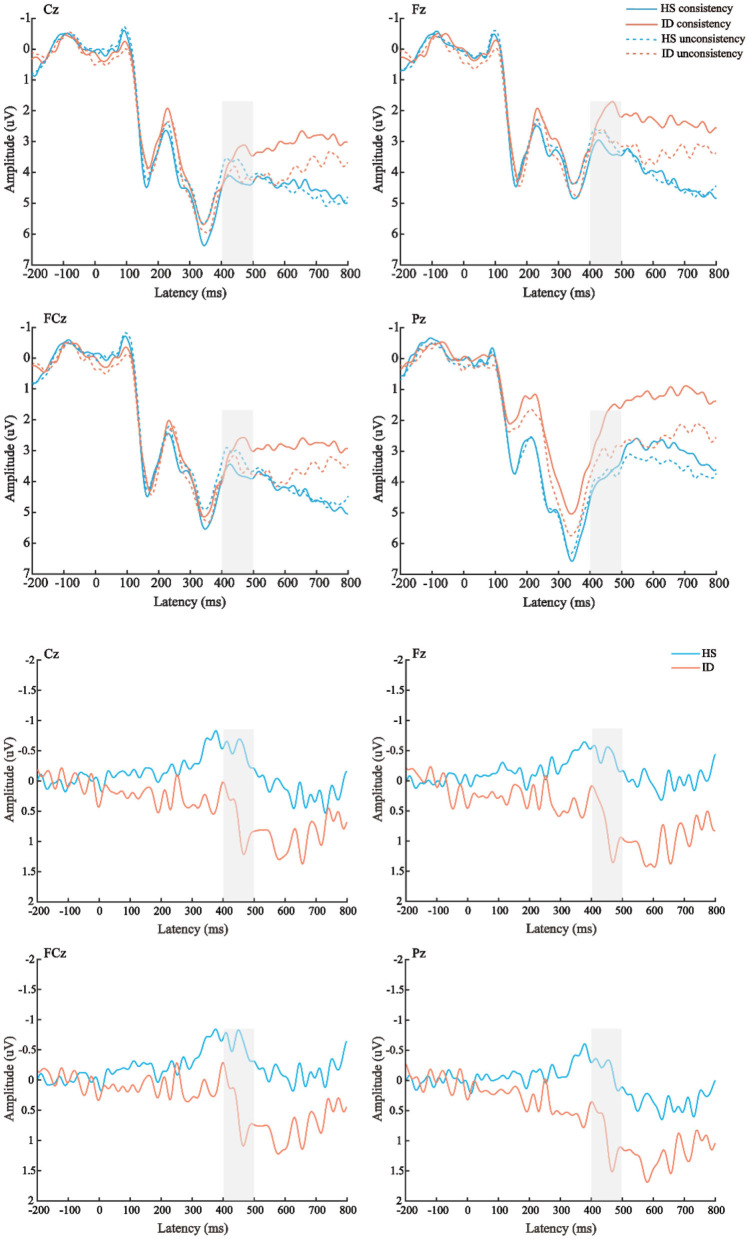
**Top**: The average ERP waveforms of the scalp positions of Cz, Fz, FCz, and Pz in the HS group and the ID group under the two conditions, respectively. **Bottom**: The mean ERP amplitudes of difference waves (inconsistent condition minus consistent condition) in Cz, Fz, FCz, and Pz scalp positions of subjects in the HS group and ID group.

The average amplitudes of the N450 components were analyzed by 2 (conditions: consistent conditions and inconsistent conditions) × 2 (groups: HS group and ID group) × 4 (electrode points: Fz, FCz, Cz, and Pz) mixed measures analysis of variance. The results are shown in Figure 7; the main effect of the electrode points was significant [*F*_(3, 180)_ = 13.501, *p* < 0.001, η2 p = 0.184], and the amplitude of the Cz electrode point (*M* ± *SE*: 3.94 ± 0.40 μV) was significantly larger than that of the FCz electrode point (*M* ± *SE*: 3.38 ± 0.41 μV), Pz electrode points (*M* ± *SE*: 3.32 ± 0.38 μV), and the amplitudes of the Fz electrode points (*M* ± *SE*: 2.82 ± 0.42 μV). Neither the main effect of the conditions [*F*_(1, 60)_ = 0.279, *p* = 0.600] nor the main effect of the groups were significant [*F*_(1, 60)_ = 0.508, *p* = 0.479].

Furthermore, the interaction between the electrode points and the groups was significant [*F*_(3, 180)_ = 3.433, *p* = 0.039, η2 p = 0.054]. A further simple effect analysis showed that the average amplitudes of the HS group at the Fz electrode point (*M* ± *SE*: 3.10 ± 0.61 μV) were greater than that of the ID group (*M* ± *SE*: 2.54 ± 0.59 μV), and the average amplitudes of the HS group at the FCz electrode point (*M* ± *SE*: 3.50 ± 0.61 μV) were greater than that of the ID group (*M* ± *SE*: 3.27 ± 0.57 μV), the average amplitudes of the HS group at the Cz electrode point (*M* ± *SE*: 4.05 ± 0.58 μV) were greater than that of the ID group (*M* ± *SE*: 3.83 ± 0.56 μV), and the average amplitudes of HS group at the Pz electrode point (*M* ± *SE*: 3.93 ± 0.54 μV) were greater than that of the ID group (*M* ± *SE*: 2.72 ± 0.53 μV).

**Figure 8:**
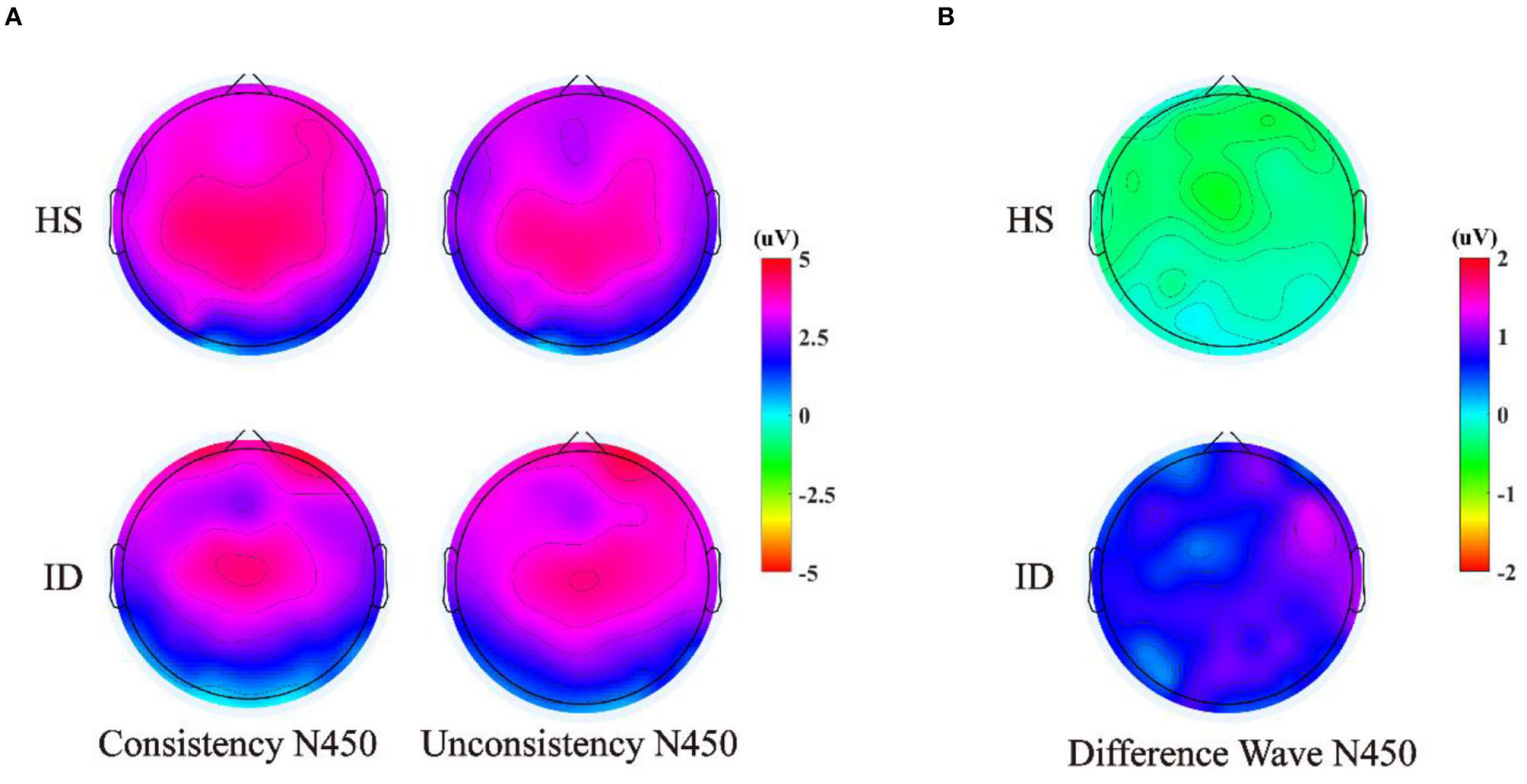
**(A)** Scalp voltage topography of N450 components in the HS group and the ID group under two conditions. **(B)** Scalp voltage topography of the difference wave in the HS group and ID group under two conditions.

The 2 (groups) × 4 (electrode points) ANOVA was performed on the N450 difference wave, and the results are shown in Figure 8. The main effect of the electrode points was significant [*F*_(3, 180)_ = 5.004, *p* = 0.013, η2 p = 0.077]. The main effect of the groups was significant [*F*_(1, 60)_ = 4.124, *p* = 0.047, η2 p = 0.064]. The amplitudes of the HS subjects (*M* ± *SE*: −0.41 ± 0.39 μV) were greater than the ID subjects (*M* ± *SE*: 0.69 ± 0.38 μV). However, there was no significant interaction between the electrode points and the groups [*F*_(3, 180)_ = 0.054, *p* = 0.921, η2 p = 0.001].

## 4 Discussion

In this study, we used ERP technology to investigate the EEG characteristics of inhibitory control in insomnia disorders (experimental group) and healthy sleepers (control group) through the two paradigms of two-choice oddball and color-word Stroop to investigate the response inhibition and conflict inhibition in the inhibitory control. The research study found that compared to the HS group, the ID group showed slower reaction times and lower correct rates, indicating weak inhibitory control. The ID group also showed lower P3 and N450 amplitudes, potentially suggesting that IDs' inhibitory control was disrupted. This study has crucial implications for people struggling with insomnia, such as impaired inhibitory control, and shows that difficulty suppressing thoughts unrelated to sleep can cause many problems for IDs. Importantly, this study also provides a theoretical supplement for the clinical treatment of IDs' inhibitory control.

### 4.1 The relationship between insomnia disorder and response inhibition

The results of the scale showed that compared to the HS subjects, the ID subjects had poorer sleep quality in daily life and may feel more fatigue during the day. This shows that ID subjects are in a state of fatigue for a long time and need to pay attention to rest and not be too stressed. In the two-choice oddball task, the behavioral results showed that under the standard stimulus condition, the correct rate of the subjects in the HS group was significantly higher than that in the ID group. The average reaction time of the subjects in the ID group was slower than that of the HS group. These results suggest that participants in the ID group may need to expend more time and effort to achieve optimal performance. This is perfectly consistent with the psychobiological inhibition/attention-intention-effort theoretical models of insomnia. Interestingly, ID subjects showed delayed reaction times not only to abnormal stimuli but also to standard stimuli, suggesting that behavioral reaction times are often disrupted in ID subjects. These results are consistent with a previous study that showed longer response times to inhibitory controls in the ID group compared to the HS group (44). Zhao et al. (45) explored response inhibition deficits in individuals with insomnia by using the stop signal task and also found that compared to healthy sleepers, ID people showed significantly longer stop signal reaction time (SSRT), suggesting that insomnia patients have impaired motor inhibition.

The display of the ERP result of the TCOT showed that there was no significant difference between the two groups in the N2 amplitude. Regarding the P3 amplitude, this study found that the deviant stimulus induced a larger P3 amplitude than the standard stimulus. The amplitudes of the Fz, FCz, Pz, and Cz electrode points of the HS group subjects were larger than those of the ID group subjects. However, these amplitude reductions may result from a decrease in generalized arousal of the brain and nervous system (46, 47). This potential confound may also be a limitation of the analysis of raw ERP values (non-difference wave). Previous studies have believed that the P3 component in the inhibitory control task reflects motor inhibition ability (27). Some studies also believe that the greater the amplitude of the P3 difference wave, the stronger the individual's motor inhibition ability may be (25, 26). The P3 amplitude was the largest in the frontal hub, which is consistent with the ERP component of the labeled response inhibition index in the oddball paradigm (48, 49). Neuroimaging studies have shown functional or structural changes in the prefrontal cortex in patients with insomnia, including reduced size, hypometabolism, and reduced activity, which can lead to impaired executive control functions dependent on this region (50, 51).

### 4.2 The relationship between insomnia disorder and conflict inhibition

The behavioral results of the color-word task showed that the average correct rate of the subjects in the HS group was higher than that in the ID group. The average reaction time of the HS group was faster than that of the ID group. The correct rate and reaction time of the subjects under the consistent condition were higher and faster than those under the inconsistent condition. This is consistent with the results of previous studies. Several studies have shown that individuals respond faster and have higher correctness rates in consistent trials compared with inconsistent trials (52–54). The average reaction time of subjects in the HS group was faster than that of the ID group in both the congruent and incongruent conditions. These results indicated that the subjects in the HS group showed a less interference effect than those in the ID group, which indicated that the ID subjects had a poor conflict inhibition ability, and their conflict inhibition ability could be abnormal. The findings support the idea proposed in the maintenance cognitive model of insomnia, in which people with insomnia try to control or switch off uncontrollable, unpleasant presleep thoughts through inhibition.

The ERP results of CWST showed that the amplitude of the N450 difference wave induced by the HS group was significantly larger than that of the ID group, which indicated that participants in the ID group may have a poorer conflict monitoring ability. Previous studies have suggested that the N450 difference wave may originate from the anterior cingulate gyrus and is related to conflict monitoring (33, 55). The greater the amplitude of the difference wave of N450, the stronger the ability to monitor conflicts may be reflected (56, 57). For example, research on the development of conflict inhibition found that older adults have diminished conflict-monitoring abilities, as evidenced by reduced behavioral performance on the Stroop task and reduced N450 differential wave amplitude (33, 58). A research study on physical training has found that compared to elderly people who lack exercise, elderly people who participate in active sports have higher accuracy, shorter reaction times, and larger N450 amplitudes when completing the Stroop task (31). Therefore, similar to previous studies, the weaker N450 differential wave of the participant ID group may reflect their poorer conflict inhibition ability. From a pathophysiological perspective, hyperactivity at the cognitive, emotional, behavioral, and autonomic or central nervous system levels in individuals with insomnia also interferes with the patient's ability to initiate or maintain sleep and leads to impaired daytime functioning (59).

In this study, the ID group was found to show elevated levels of fatigue and anxiety symptoms compared to the HS group, which was consistent with previous reports showing an association between insomnia and the risk of psychopathology (60). Insomnia severity increases with anxiety symptoms in individuals with emotional regulation difficulties, while changes in anxiety symptoms are not affected by the severity of insomnia symptoms (61). Increased rumination, a strong cognitive risk factor for depression, was associated with specific changes in both N2 and P3 amplitudes in reaction to non-emotional faces in girls of mothers with histories of depression according to a previous study that evaluated ERP during an emotional go/no-go task (62). Inhibitory control deficits can play a role in cognitive processes associated with the risk of depression and anxiety in individuals with insomnia. As this study involves the multidimensional relationship between sleep and psychopathology, further longitudinal research is required to better explore the neurocognitive mechanisms underlying the relationship between insomnia and psychopathology.

Our current study has some limitations. First, this study did not balance the gender of the participants. Future studies could examine whether there are sex differences in impaired inhibitory control in individuals with ID. Second, although we excluded patients with anxiety, negative emotions may influence individuals' behavioral inhibition. Posner et al. (63) showed that negative emotions have an interfering effect on individual behavioral inhibition. Hartikainen et al. (64) used the go/no-go paradigm to induce individual fear emotions with fearful pictures, such as spiders. The results showed that the reaction time of individuals with fear was longer than that of individuals with neutral conditions; that is, fear can hinder behavioral inhibition. Finally, no measure of vigilance was taken during the experiment. Some studies have found that patients with insomnia have lower accuracy and slower speed in maintaining attention tasks (65, 66). Other studies have explored the relationship between sleep problems and attention deficit and found that the degree of inattention was significantly related to the severity and duration of sleep problems (67). Therefore, part of the reason for slower reaction times in IDs may be a lack of sleep/attention deficit.

## 5 Conclusion

Adults with insomnia demonstrated altered brain activity during inhibitory control, and IDs have worse behavioral performance. In general, this suggests that insomnia may affect an individual's inhibitory control. Future studies with a longitudinal design could further explore the long-term impacts and trajectory of altered inhibitory control in adults with insomnia.

## Data availability statement

The original contributions presented in the study are included in the article/supplementary material, further inquiries can be directed to the corresponding authors.

## Ethics statement

The studies involving humans were approved by Scientific Research Ethics Committee of the School of Psychology, Northwest Normal University. The studies were conducted in accordance with the local legislation and institutional requirements. The participants provided their written informed consent to participate in this study.

## Author contributions

XD: Conceptualization, Project administration, Resources, Supervision, Validation, Writing—review & editing. LH: Conceptualization, Formal analysis, Investigation, Methodology, Software, Writing—original draft, Writing—review & editing. XG: Data curation, Formal analysis, Investigation, Writing—original draft. XZhao: Investigation, Methodology, Validation, Writing—original draft. ZH: Investigation, Methodology, Software, Writing—original draft. XZhan: Conceptualization, Data curation, Funding acquisition, Project administration, Resources, Writing—review & editing.
